# Enhanced Antitumor Efficacy of a Combination of Immunotoxin and Photosensitizer Under Illumination in Xenograft Mice

**DOI:** 10.3390/biomedicines14030573

**Published:** 2026-03-03

**Authors:** Shunji Hamakubo, Noriko Komatsu, Azuma Kosai, Mikako Kuroda, Masataka Sawada, Reina Shimizu, Riuko Ohashi, Hideyuki Suenaga, Takao Hamakubo, Takahiro Abe

**Affiliations:** 1 Department of Oral and Maxillofacial Surgery, Kanagawa Dental University, Yokosuka 238-8570, Japan; 2 Department of Oral-Maxillofacial Surgery and Orthodontics, The University of Tokyo, Tokyo 113-8655, Japan; 3 Histopathology Core Facility, Faculty of Medicine, Niigata University, Niigata 951-8510, Japan; 4 Division of Molecular and Diagnostic Pathology, Graduate School of Medical and Dental Sciences, Niigata University, Niigata 951-8510, Japan; 5 PhotoQ3 Inc., Tokyo 102-0084, Japan; 6 Department of Oral and Maxillofacial Surgery, Second Department of General Medicine, Jichi Medical University Saitama Medical Center, Saitama 330-8503, Japan

**Keywords:** immunotoxin, photodynamic therapy (PDT), head and neck squamous cell carcinoma, saporin, cetuximab, photosensitizer, intelligent targeted antibody phototherapy (iTAP)

## Abstract

**Background/Objectives**: Head and neck squamous cell carcinoma (HNSCC) affects over 600,000 individuals worldwide each year, and its incidence continues to rise. There is a growing need for novel therapeutic strategies that achieve high antitumor efficacy while minimizing functional impairment. We developed a novel approach to enhance intracellular delivery of immunotoxins (ITs) by combining a photosensitizer under illumination. This method, termed intelligent Targeted Anti-body Phototherapy (iTAP), utilizes light as a spatiotemporal trigger to promote the cytoplasmic release of toxins. In the present study, we investigated the in vivo therapeutic efficacy of iTAP using an EGFR-targeted IT composed of cetuximab conjugated to saporin (IT-Cmab), administered in combination with the clinically used photodynamic therapy (PDT) photosensitizer NPe6, in a xenograft mouse model. **Methods**: Sa3 cells were implanted subcutaneously into the right hind limb of nude mice. Mice were randomized into four groups (n = 5): (i) iTAP (IT-Cmab plus NPe6), (ii) IT-Cmab alone, (iii) NPe6 alone, and (iv) saline control. Treatment was initiated once tumors exceeded 40 mm^3^. Mice received intraperitoneal IT-Cmab (0.5 mg/kg), followed 72 h later by intravenous NPe6 (5 mg/kg). Tumors were irradiated 3–4 h later using a custom LED device (670 nm, 262 mW/cm^2^, 30 J/cm^2^). Tumor volume and body weight were monitored over time, and antitumor effects were analyzed using a linear mixed-effects model. **Results**: iTAP treatment produced the earliest and most pronounced inhibition of tumor growth among the four groups. Significant suppression was observed from day 9 and persisted throughout the study. IT-Cmab alone showed a moderate but sustained antitumor effect with a later onset, whereas NPe6-mediated PDT exhibited only a delayed and weaker response. On the final day, median tumor volumes showed substantial reductions relative to the Control group (601%), with decreases of 41% in PDT (357%), 55% in IT-Cmab (271%), and 70% in iTAP (178%). Overall, iTAP demonstrated the strongest and most durable therapeutic efficacy in vivo. **Conclusions**: These findings indicate that iTAP represents a promising therapeutic strategy for HNSCC.

## 1. Introduction

Head and neck cancer has been reported to occur in approximately 660,000 cases annually worldwide, with 325,000 deaths [1,2]. Although the etiological factors of cancer have shifted over time, the incidence continues to rise, representing a significant social problem [3]. The standard treatment for head and neck squamous cell carcinoma consists of a combination of surgery, chemotherapy, and radiotherapy. Various complications, however, including difficulties with eating, speech, breathing, and physical appearance, ultimately compromise patients’ quality of life (QOL) [4]. Minimizing such complications is essential. Antibody-based therapeutics, such as anti-programmed death-1 (PD-1) agents and the epidermal growth factor receptor (EGFR)-targeting monoclonal antibody cetuximab, are associated with fewer adverse effects, but their efficacy against solid tumors remains limited [5].

In recent years, a variety of antibody–drug conjugates (ADCs) have been developed to enhance the antitumor efficacy of therapeutic antibodies by conjugating potent cytotoxic payloads to cancer-specific antigens such as EGFR or HER2 via specialized linkers. However, increasing evidence suggests that payload-associated toxicities and the immunosuppressive nature of the tumor microenvironment may limit the therapeutic effectiveness of ADCs [6].

In addition to *Pseudomonas* exotoxin, plant-derived ribosome-inactivating toxins such as saporin have been developed as payloads for ITs [7,8]. Saporin is a protein toxin (molecular weight ~25 kDa) classified as a type 1 ribosome-inactivating protein and is derived from the seeds of *Saponaria officinalis*. Although it cannot enter cells on its own, once internalized, it irreversibly inactivates ribosomes by its N-glycosidase activity, thereby inhibiting protein synthesis and inducing cell death. When conjugated to tumor-targeting antibodies, saporin confers selective cytotoxicity toward antigen-expressing cancer cells [9,10].

In another vein, photodynamic therapy (PDT), which uses a combination of a photosensitizing dye and a specific light source, has attracted attention as a non-invasive antitumor treatment [11]. PDT involves the activation of a photosensitizer by light of a specific wavelength, generating reactive oxygen species such as singlet oxygen that induce oxidative damage to cellular structures and lead to apoptosis or necrosis. PDT also contributes to tumor control by disrupting tumor vasculature and enhancing antitumor immune responses. Among several photosensitive dyes developed such as porfimer sodium (Photofrin^®^; 630 nm) or verteporfin (Visudine^®^; 689 nm), mono-L-aspartyl chlorin e6 (NPe6, talaporfin sodium, Laserphyrin^®^, maximum absorption peak at 664 nm) is approved for early stage lung cancer, malignant brain tumors, and local recurrent esophageal cancer in Japan [12]. In HNSCC, various PDT approaches have been investigated. However, despite their therapeutic potential, limitations in photosensitizer delivery and light penetration persist, and PDT has not yet become an established treatment modality for HNSCC [13]. To address this limitation, we developed a novel therapeutic strategy that enhances efficacy by combining PDT with an IT that offers superior cytotoxic potency compared to conventional ADCs. We have designated this synergistic method as intelligent Targeted Antibody Phototherapy (iTAP) [14,15].

Our previous in vitro studies demonstrated that among several HNSCC-derived cell lines, Sa3 exhibited the highest EGFR expression and showed the strongest iTAP response [15]. This study demonstrated the iTAP effect in HNSCC in vivo using a mouse xenograftmodel through the combination of an IT consisting of anti-EGFR antibody (cetuximab), conjugated to saporin (IT-Cmab) and NPe6-based PDT.

In this study, the interval between IT administration and light irradiation was set to 3 days. This timing was chosen based on our previous experience and on reports from other investigators, including PET imaging studies in both mice and humans [16,17], demonstrating that systemically administered antibodies typically reach peak intratumoral accumulation approximately 2–4 days after injection [14]. Therefore, the treatment schedule was intentionally designed to coincide with the expected maximal antibody concentration within the tumor, which is considered critical for achieving the optimal therapeutic effect of iTAP.

To ensure homogeneous irradiation of the tumor area, a custom-built LED irradiation system was developed and employed. Results suggest that iTAP using IT-Cmab is effective against HNSCC and may serve as a potential therapeutic modality

## 2. Materials and Methods

### 2.1. Cells

The HNSCC cell line Sa3 (derived from human oral cancer, RCB0980) was purchased from RIKEN (Tsukuba, Japan). Sa3 cells were cultured in Basal Medium Eagle (BME; Gibco, Thermo Scientific, Waltham, MA, USA) supplemented with 1% L-Alanyl-L-Glutamine Solution (Wako, Tokyo, Japan), 1% antibiotics (Penicillin-Streptomycin Solution, Wako, Tokyo, Japan), and 20% Newborn Calf Serum (Gibco, Thermo Scientific, MA, USA). Cells were incubated at 37 °C in a humidified atmosphere containing 5% CO_2_. Dulbecco’s phosphate-buffered saline (D-PBS) was purchased from Wako (Tokyo, Japan).

### 2.2. Chemicals

#### 2.2.1. Immunotoxin

Anti-EGFR antibody (cetuximab; Cmab) was purchased from Merck Serono (Darmstadt, Germany). IT was prepared as previously described [15]. First, Cmab was biotinylated using EZ-LINK sulfo-NHS-LC-biotin (Thermo Fisher Scientific, Waltham, MA, USA) at a 1:20 molar ratio. After purification using PD-10 columns (GE Healthcare Life Sciences, Piscataway, NJ, USA), the number of conjugated biotin molecules per antibody was estimated to be approximately 8 using a HABA assay kit (AnaSpec, Fremont, CA, USA) based on absorbance measurements at 500 nm [18]. Biotinylated Cmab was mixed with streptavidin-saporin (Biotin-Z Internalization Kit (KIT-27-Z), Advanced Targeting Systems, Carlsbad, CA, USA) in approximately equivalent amounts to react at room temperature for 30 min. Obtained IT is called IT-Cmab hereafter. The formation of the conjugated product was assessed by a sandwich ELISA. An anti-Erbitux idiotype antibody was immobilized as the capture antibody, and bound conjugates were detected using an anti-saporin antibody (Supplementary Figure S1).

#### 2.2.2. Photosensitizer

NPe6 was purchased from Meiji Seika Pharma (Tokyo, Japan). NPe6 was dissolved in sterilized saline (Otsuka, Tokushima, Japan) at 25 mg/mL, and stored at −35 °C in aliquots. NPe6 was further diluted to 0.5 mg/mL with saline before use, and 200 μL was administered intra-venously.

### 2.3. Photochemical Equipment

**Figure 1:**
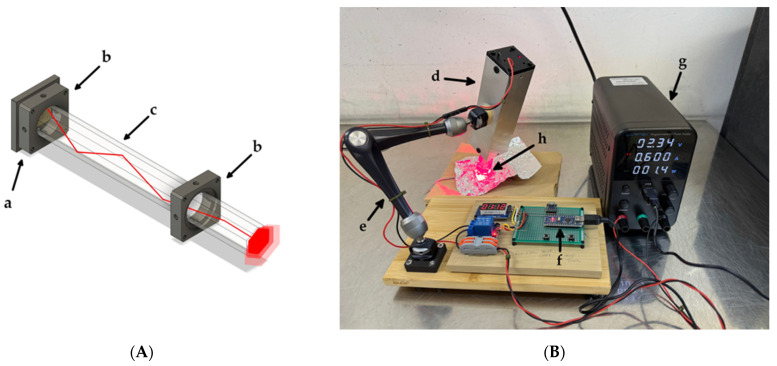
Custom LED irradiation system. (**A**) LED assembly. (a) LED light source. (b) Support plate. (c) Hexagonal glass rod. (**B**) LED irradiation device. (d) Aluminum extrusion housing. (e) Articulating bracket. (f) Arduino Nano microcontroller. (g) Power supply. (h) Irradiation of the tumor site shielded with aluminum foil.

The illuminator was constructed in-house with an emphasis on irradiation homogeneity, high radiance, and effective thermal isolation from the light source. An LED light source coupled to a glass light rod was selected for its simplicity of alignment and its ability to deliver stable irradiance while maintaining high spatial homogeneity across the irradiation field [19]. A single 670-nm light-emitting diode (SMBB670D-1100; Ushio Inc., Tokyo, Japan) was coupled to one end of a hexagonal glass light rod (20 × 200 mm; #84-536, Edmund Optics, Barrington, NJ, USA) (Figure 1A). The assembly was housed within a stock aluminum extrusion at hand and supported by an articulating bracket (3959, SmallRig, Shenzhen, China), allowing for precise positioning and stable fixation of the illuminator relative to the animal. The LED was operated in constant-current mode at 600 mA using a DC power supply. Irradiation duration was controlled by an Arduino Nano microcontroller (Arduino S.r.l., Monza, Italy) (Figure 1B). Irradiance was measured using a calibrated optical power meter (Model 8230E with an 82313 sensor wand; ADCMT, Tokyo, Japan).

### 2.4. Animals

All procedures involving mice were carried out in accordance with the protocols approved by the Institutional Animal Care and Use Committee at Kanagawa Dental University (Approval number 25-003). Male BALB/cSlc-nu/nu mice of 8 weeks old (21–25 g) were used.

Semiconfluent Sa3 cells cultured in 15 cm dishes were dissociated by 0.25% Trypsin EDTA-4Na Solution (Wako, Tokyo, Japan), washed twice and centrifuged. The cells were suspended in D-PBS and adjusted to a density of 1 × 10^8^ cells/100 μL. Equal amounts of cell suspension and basement membrane matrix gel (Matrigel; Corning, Corning, NY, USA) were mixed.

Sa3 tumor cells, 1 × 10^8^/200 μL, were inoculated subcutaneously into the right proximal hindlimb region of each mouse under peritoneal anesthesia of a mixture of medetomidine, midazolam, and butorphanol (MMB). Water and food were provided ad libitum.

To prevent excessive light exposure, the cages of all mice were covered with green transparent cellophane immediately after irradiation, and shading was maintained throughout the observation period.

The growth of the tumor was monitored by measuring the tumor size every 1 to 2 days. The tumor size was calculated using the following formula [20]:

V = (length × width^2^)/2

where length is the longest diameter and width is the shortest diameter. At each tumor-volume measurement, photographs of both the tumor site and the whole body were taken for all mice, and the images were reviewed independently by multiple observers.

The experiments were initiated when the tumor size reached 40 mm^3^. Mice were randomly divided into four groups (n = 5) as follows: (i) iTAP: 0.5 mg/kg IT-Cmab in 200 μL saline i.p. plus 5 mg/kg NPe6 in 200 μL saline i.v.; (ii) IT-Cmab only: 0.5 mg/kg IT-Cmab in 200 μL saline i.p. plus 200 μL saline i.v.; (iii) NPe6 only (PDT): 200 μL saline i.p. plus 5 mg/kg NPe6 in 200 μL saline i.v.; and (iv) Saline control: 200 μL saline i.p. plus 200 μL saline i.v. IT-Cmab was administered on day 0 and NPe6 was injected from the tail vein on day 3. Three to four hours after NPe6 injection, the tumors were illuminated with 670 nm light using a custom LED device under MMB anesthesia. The mouse leg was covered with aluminum foil that had an opening for the tumor with a 2–3 mm free margin, to which the light was exposed (Figure 1B). During PDT, the animals’ respiration and general condition were continuously observed by the experimenter. An antagonist for medetomidine (0.75 mg/kg, intraperitoneally) was administered immediately after the procedure to ensure safe and prompt recovery from anesthesia. No abnormalities were observed during or after the procedure. All of the animals received light illumination at the tumor as described above. When the size of the tumor reached 20 mm in diameter or the body weight of the mice decreased drastically (loss of more than 20% in a week), the mice were euthanized according to institutional guidelines. In the pre-test using engraft mice, tumor masses exceeding 20 mm were observed after day 30; therefore, the main experiment was terminated at day 30.

### 2.5. Data Analysis

Data are shown as the means ± S.E. Statistical evaluation was performed using analysis of linear mixed model. Differences with a value *p <* 0.01 were taken to be statistically significant.

## 3. Results

### 3.1. Light Irradiation

Using the custom illuminator described in the Materials and Methods section, the irradiance at the distal end of the light rod was 262.3 mW/cm^2^ when driven at 600 mA. During the experiments, the end of the light rod opposite the LED was placed in direct contact with the implanted tumor region (Figure 1B). When delivering a total light dose of 30 J/cm^2^ (114.4 s), the tissue-contacting end of the rod did not become perceptibly warmer than ambient temperature, as assessed by direct fingertip palpation. The results of a supplementary thermographic assessment using a white polystyrene block under the same illumination conditions are shown (Supplementary Figure S2). This was attributable to the 200-mm separation from the heat-generating LED and the thermal capacity of the assembly.

### 3.2. Animal Weight

**Figure 2:**
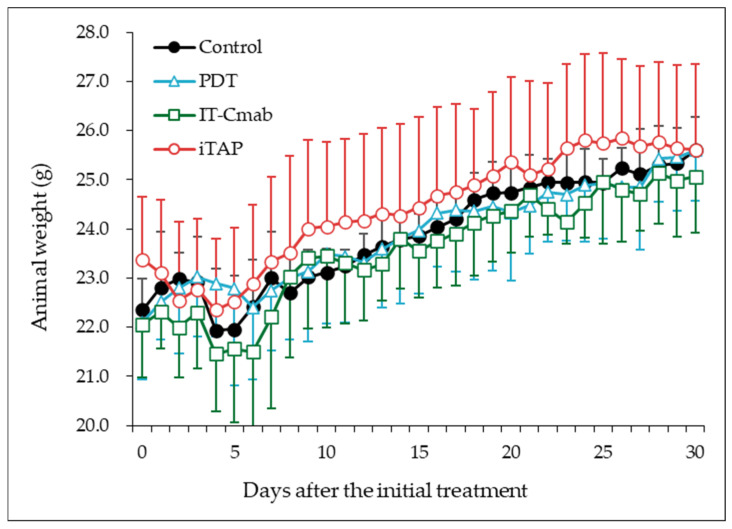
Animal weight changes after different treatments. (i) iTAP (open circle): 0.5 mg/kg IT-Cmab i.p. plus 5 mg/kg NPe6 in 200 μL saline i.v.; (ii) IT-Cmab only (open square): 0.5 mg/kg IT-Cmab i.p. plus 200 μL saline i.v.; (iii) NPe6 only (PDT; open triangle): 200 μL saline i.p. plus 5 mg/kg NPe6 in 200 μL saline i.v.; and (iv) Saline control (closed circle): 200 μL saline i.p. plus 200 μL saline i.v. Data are shown as the mean ± S.E. No significant differences were observed among the groups (analysis of linear mixed model, p > 0.2).

With respect to body weight changes, a slight decrease was observed in all groups on days 4–6 after the initial treatment. Thereafter, a gradual increase was noted, and no statistically significant differences were detected among the groups (*p* > 0.2) (Figure 2).

### 3.3. Tumor Volume

In all mice, no edema or pathological skin changes were observed outside the irradiation site. In the iTAP and PDT groups, edema at the irradiation site was noted from the day following light exposure but resolved within several days. In the iTAP group, ulcer formation was observed in one out of five mice beginning on day 6 after irradiation, followed by eschar formation within a few days, which subsequently progressed to complete healing.

**Figure 3:**
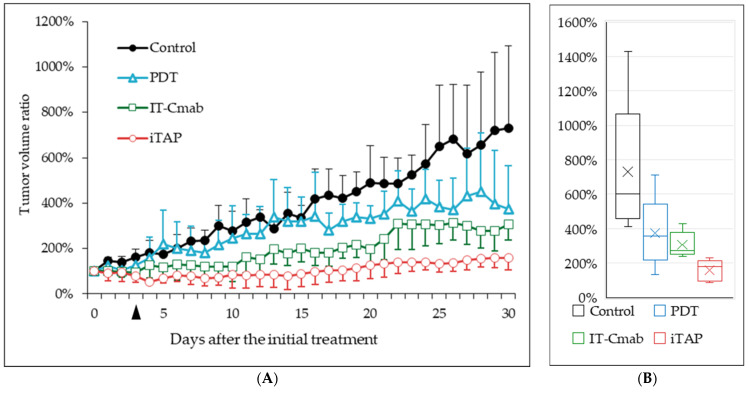
(**A**) Tumor growth curve of Sa3 tumor-bearing mice after different treatments. (i) iTAP (open circle): 0.5 mg/kg IT-Cmab i.p. plus 5 mg/kg NPe6 in 200 μL saline i.v.; (ii) IT-Cmab only (open square): 0.5 mg/kg IT-Cmab i.p. plus 200 μL saline i.v.; (iii) NPe6 only (PDT; open triangle): 200 μL saline i.p. plus 5 mg/kg NPe6 in 200 μL saline i.v.; and (iv) Saline control (closed circle): 200 μL saline i.p. plus 200 μL saline i.v. Data are shown as the mean ± S.E. In the iTAP group, tumor volume growth was significantly suppressed compared with the IT-Cmab monotherapy, PDT monotherapy, and saline control groups (analysis of linear mixed model, *p* < 0.0001). The day on which NPe6 or saline was administered and light irradiation was performed are indicated by arrowhead. (**B**) shows a comparison of tumor volumes on day 30 among the four groups using box-and-whisker plots. Box-and-whisker plots display the median (horizontal line), interquartile range (box), and range (whiskers). Mean values are indicated by cross marks (×).

We evaluated the iTAP effect by randomly assigning mice to four treatment groups, (i) iTAP, (ii) IT-Cmab only, (iii) NPe6 only (PDT), and (iv) Saline control, as described in the Materials and Methods section. In all mice, tumors grew to approximately 40 mm^3^ within 2–3 days after the inoculation of Sa3 cells mixed with Matrigel into the right proximal hindlimb region. Tumor volume was expressed as a percentage, with the start date of the experiment set as 100%. Tumor volumes increased progressively over the 30-day observation period in all groups (Figure 3A). A linear mixed-effects model was applied to evaluate longitudinal differences in tumor growth among treatment groups. The model included Group, Day, and their interaction (Group × Day) as fixed effects, and a random intercept for each mouse.

Progressive tumor enlargement over time was observed (day 9 onward, all *p* < 0.01). A significant Group × Day interaction emerged across multiple time points, demonstrating that tumor growth trajectories differed among treatment groups. The iTAP treatment exhibited the earliest and most pronounced inhibitory effect on tumor growth. Significant suppression relative to the control group was observed beginning on day 9 (*p* = 0.030) and persisted consistently through day 30 (all *p* < 0.001). This indicates a robust and sustained antitumor effect. The IT-Cmab group showed a moderate but clear treatment effect. Significant reductions in tumor growth appeared from day 16 (*p* = 0.024) and continued through the late phase of the study (day 30, *p* < 0.001). The onset of effect was later than iTAP but remained statistically meaningful thereafter. The PDT group demonstrated a delayed therapeutic response. Significant differences were first detected on day 25 (*p* = 0.011), with sustained significant suppression observed (*p* < 0.01). Although weaker than iTAP and IT-Cmab, PDT still exerted measurable antitumor activity in the late phase.

Tumor volumes on the final day of the experiment were compared among the four groups and are presented as box-and-whisker plots in Figure 3B. The median values differed substantially among the four groups: Control (601%), PDT (357%), IT-Cmab (271%), and iTAP (178%). The Control group showed the highest median, whereas iTAP exhibited the lowest. The median difference between Control and PDT was 244%, and the difference between Control and iTAP reached 423%.

Together, these findings indicate that iTAP provides the strongest and earliest inhibition of tumor progression, followed by IT-Cmab with a moderate effect, while PDT shows a detectable antitumor response.

## 4. Discussion

Various light sources have been used in PDT, including lasers, LEDs, and incandescent lamps, each with distinct advantages [11]. Lasers offer high power and precise wavelength control but irradiate only small areas and require costly, technically demanding equipment. LEDs, although lower in power, are inexpensive, easy to handle, and capable of illuminating broader or curved surfaces when arranged in arrays, as demonstrated in metronomic PDT [21]. However, LEDs often exhibit broad spectra, large beam divergence, and heat-related issues.

Previous studies using LED lamps required tens of minutes to deliver a comparable light dose [15]. In contrast, our system in this study achieved the target fluence in less than two minutes by transmitting LED light through a glass light rod, which reduced beam divergence and minimized energy loss. Importantly, this rod-type light source can be bundled into an array configuration, enabling the illumination of a wider area while suppressing heat generation. A limitation of this study is the absence of direct, quantitative temperature monitoring during illumination, which prevents a definitive assessment of heat-related effects. These characteristics suggest that the device may be suitable for treating larger tumors, where uniform irradiation is essential. Further miniaturization and array configuration of this device may enable uniform irradiation tailored to the complex geometry of head and neck tumors.

Overexpression of EGFR is observed in a substantial proportion (40–80%) of HNSCC cases, and the anti-EGFR antibody cetuximab is approved for the treatment of HNSCC [22]. However, cetuximab monotherapy frequently leads to the development of antibody resistance, resulting in limited antitumor efficacy. Consequently, combination strategies—such as cetuximab with radiotherapy [23], or with immune checkpoint inhibitors [24]—have been investigated. However, the therapeutic effect remains insufficient.

In recent years, ADCs incorporating cytotoxic payloads into anti-HER2 antibodies have demonstrated high efficacy in breast cancer, generating considerable interest. In HNSCC, multiple clinical trials are ongoing to evaluate ADCs targeting EGFR, HER2, Trop-2, and Tissue Factor, conjugated with small-molecule cytotoxins such as MMAE; however, severe adverse effects have limited their clinical progress [25].

**Figure 4:**
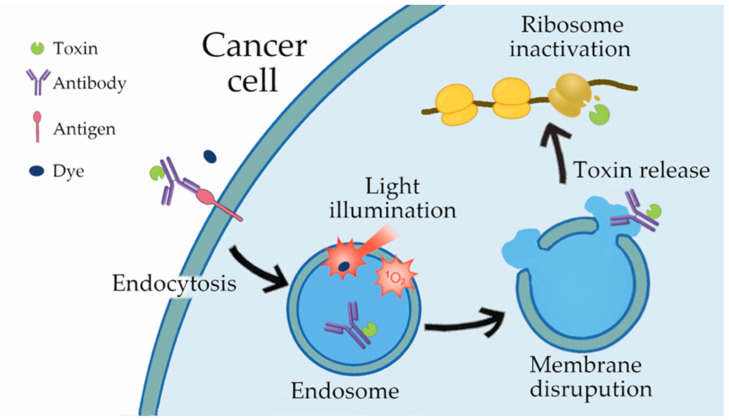
The mechanism of action of the iTAP approach is illustrated as follows. Upon binding of the immunotoxin to its cognate antigen on the cancer cell surface, the complex is internalized through receptor-mediated endocytosis. Separately, administration of a photosensitizing dye followed by light irradiation generates singlet oxygen (^1^O_2_), which disrupts the endosomal membrane and enables cytosolic release of the toxin. The liberated toxin subsequently inactivates ribosomes, leading to the inhibition of protein synthesis and ultimately inducing cell death.

ITs represent a subclass of ADCs in which the payload consists of enzymatic protein toxins, and they have been reported to exert stronger antitumor activity than ADCs carrying small-molecule drugs [7]. Nevertheless, ITs are also associated with substantial toxicity, restricting the doses that can be safely administered [26]. In this regard, the iTAP approach enhances the therapeutic effect even with low doses of IT, thereby reducing toxicity [14,15]. The mechanism of action of iTAP is illustrated in Figure 4. Although the primary target of singlet oxygen generated (^1^O_2_) during PDT is the mitochondria [11], NPe6 is known to distribute from endosomes to lysosomes [27]. iTAP exploits this reactive oxygen species to disrupt the endosomal membrane [14], thereby facilitating the cytosolic release of endocytosed immunotoxin. The liberated toxin subsequently inactivates ribosomes, thereby inhibiting protein synthesis and concurrently triggering a ribotoxic stress response, ultimately leading to cell death [28].

Importantly, the cytotoxic mechanism of the immunotoxin used in this study does not rely on the inhibition of cell division. Instead, the released toxin inactivates ribosomes and suppresses protein synthesis, thereby inducing a ribotoxic stress response that leads to cell death. Because this mechanism is independent of proliferative status, the immunotoxin is expected to exert cytotoxicity not only against rapidly dividing tumor cells but also against slowly proliferating or dormant populations, including cancer stem like cells. As clinical tumors contain heterogeneous cell populations with variable growth rates, the ability to target dormant cells is particularly important for preventing recurrence and treatment resistance. In this respect, the mechanism of iTAP differs fundamentally from conventional mitosis dependent ADCs and may offer a therapeutic advantage as a strategy capable of eliminating tumor cells that are refractory to proliferation dependent cytotoxic agents.

However, because the xenograft model used in this study predominantly reflects the behavior of rapidly dividing tumor cells shortly after implantation, it does not allow for a direct evaluation of the efficacy of iTAP against slowly proliferating or dormant tumor cell populations. This represents a limitation of the present study, and future work using models that better recapitulate tumor heterogeneity will be required to address this point.

All four mouse groups used in this study (saline control, PDT alone, IT-Cmab alone, and iTAP) exhibited a transient decrease in body weight during days 4–6, which was considered to be attributable to procedural factors such as handling and anesthesia. In the PDT and iTAP groups, some animals developed localized edema or ulceration at the irradiation site; however, these changes resolved spontaneously within several days. Edema is frequently observed as a consequence of PDT [29].

Among the evaluated therapies, iTAP showed the earliest and most pronounced deviation from the control tumor-growth trajectory. Significant separation from the control group was observed as early as day 9 and continued throughout the observation period, indicating an early onset and persistent alteration of tumor progression. In contrast, IT-Cmab demonstrated a more gradual pattern, with significant differences emerging from day 16 onward, whereas PDT exhibited the latest onset, with measurable divergence appearing only after day 25. These findings suggest that the timing and magnitude of treatment-related changes in tumor growth vary substantially across modalities, underscoring the importance of longitudinal assessment when evaluating therapeutic performance.

The differential response profiles observed here may reflect distinct mechanisms of action inherent to each therapy. iTAP’s early and sustained effect suggests efficient engagement of its target pathway and rapid disruption of tumor growth dynamics. In the IT-Cmab-only group, the administered dose was associated with a measurable but modest reduction in tumor growth. In the PDT group, early differences from the control group were not apparent, which may partly reflect transient edema at the irradiation site; however, divergence became evident during the later phase of observation.

Taken together, these findings suggest that iTAP may represent a well-tolerated and potentially useful therapeutic approach for EGFR-overexpressing HNSCC. However, this study has several limitations, including the absence of structural or functional validation of the saporin–cetuximab conjugate within this experiment and the inherent constraints of the xenograft model in capturing tumor heterogeneity. Future studies using additional models, including immune-competent mice, will be important to clarify whether iTAP induces immunogenic cell death, modulates the tumor microenvironment, and synergizes with PDT through immune activation.

## 5. Conclusions

The LED irradiation device used in this study efficiently delivered the required fluence within a short period of time, enabling practical and reproducible PDT illumination. The iTAP approach induced clear antitumor effects in the xenograft mouse model; although transient edema and ulcer formation were observed as treatment-related adverse effects, the overall findings support iTAP as a promising therapeutic strategy for EGFR-overexpressing HNSCC.

## Data Availability

Data are contained within the article and Supplementary Materials. The datasets generated during and analyzed during the current study are available from the corresponding author on reasonable.

## Supplementary Materials

The following supporting information can be downloaded at: https://www.mdpi.com/article/10.3390/biomedicines14030573/s1, Figure S1: Sandwich ELISA of IT-Cmab. Each data point represents the mean of two measurements after subtracting the fluorescence intensity of the blank (buffer only); Figure S2: A 2-cm-thick white polystyrene block was irradiated using the illumination device employed in this study under the same experimental conditions (30 J/cm^2^; 262.3 mW/cm^2^ for 114.4 s). Surface temperature was recorded using a HIKMICRO E01 thermographic camera (HIKMICRO, Hangzhou, China). At the irradiation site (indicated as “Cen” in the figure, marked by a white target mark), the maximum temperature increase observed was 1.2 °C (from 13.2 °C to 14.4 °C). Captured from a position 30 cm away from the center: (A) start point and (B) after 114.4 s.

## Abbreviations

The following abbreviations are used in this manuscript:

HNSCC	Head and neck squamous cell carcinoma
iTAP	intelligent Targeted Antibody Phototherapy
IT	Immunotoxin
PDT	Photodynamic therapy
IT-Cmab	IT composed of cetuximab conjugated with saporin
NPe6	Mono-L-aspartyl chlorin e6
MMB	Mixture of medetomidine, midazolam, and butorphanol
ADCs	Antibody–drug conjugates
