# Supplementary material for: Enhanced Antitumor Efficacy of a Combination of Immunotoxin and Photosensitizer Under Illumination in Xenograft Mice
Source: Biomedicines. 2026 Mar 3;14(3):573. doi: 10.3390/biomedicines14030573 (PMC13024140; doi:10.3390/biomedicines14030573)
Supplement: Supplementary file 1 [file biomedicines-14-00573-s001.zip › biomedicines-4129981-supplementary.pdf]

Supplementary Figure S1

Sandwich ELISA of IT-Cmab

Method

A black 96-well Immuno Plate (Thermo Fisher Scientific, MA, USA) was coated with the capture antibody, an anti-Erbitux idiotype antibody (Bio-Rad, CA, USA), at 1 µg/mL in PBS (50 µL/well), sealed, and incubated overnight at 4° C. The wells were blocked with 5% BSA/PBS (200 µL/well) for 1 h at room temperature. After five washes with PBS containing 0.05% Tween-20 (PBST), a mixture of biotinylated Erbitux and streptavidin–saporin (ATS, CA, USA) was prepared by diluting biotinylated Erbitux to a final concentration of 1 µg/mL in 40% Block Ace/PBS (BA/PBS), followed by a three-fold serial dilution in BA/PBS. The samples were added at 50 µL/well and shaken at 700 rpm for 1 h at room temperature. After five PBST washes, HRP-conjugated goat anti-saporin antibody (AB-15HRP, final 1 µg/mL) was added at 50 µL/well and shaken at 700 rpm for 1 h at room temperature. Following five additional PBST washes, the working solution of the fluorogenic substrate (QuantaBlu Fluorogenic Peroxidase Substrate Kit, Thermo Fisher Scientific, MA, USA) was prepared according to the manufacturer's instructions and added at 50 µL/well. After a 30-min incubation at room temperature, 50 µL/well of stop solution was added. Fluorescence was measured using a microplate reader (Infinite 200 PRO, Tecan Group Ltd., Zurich, Switzerland) at Ex. 320 nm and Em. 400 nm.

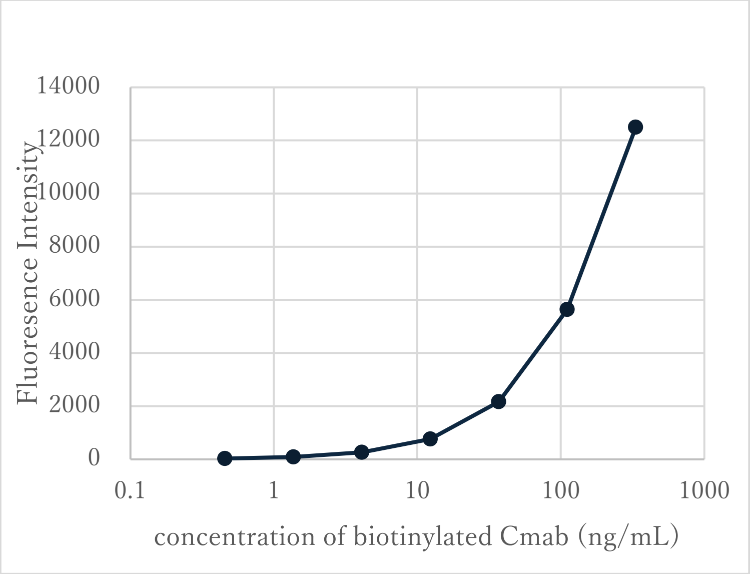

Supplementary Figure S1. Sandwich ELISA of IT-Cmab. Each data point represents the mean of two measurements after subtracting the fluorescence intensity of the blank (buffer only)

Supplementary Figure S2

Thermographic assessment of photochemical equipment used in this study

Method and Result

A 2-cm-thick white polystyrene block was irradiated using the illumination device employed in this study under the same experimental conditions (30 J/cm^2; 262.3 mW/cm^2 for 114.4 s). Surface temperature was recorded using a HIKMICRO E01 thermographic camera (HIKMICRO, Hangzhou, China). At the irradiation site (indicated as “Cen” in the figure, marked by a white target mark), the maximum temperature increase observed was 1.2 ˚C (from 13.2 ˚C to 14.4 ˚C). Captured from a position 30 cm away from the center: (A) start point and (B) after 114.4 s.

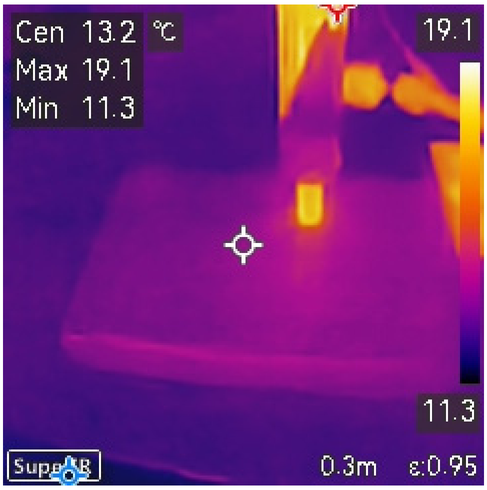

(A)

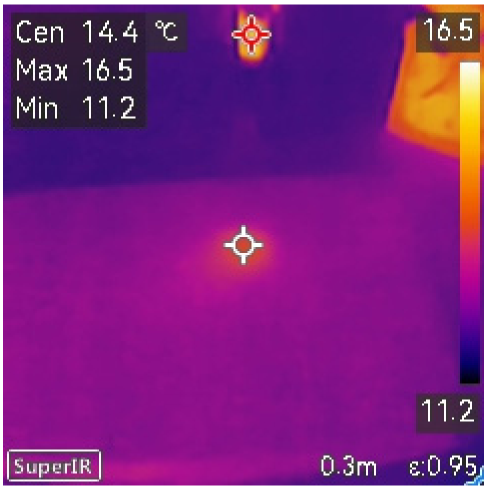

(B)
